# Impact of Nutrition or FDA-Approved Medicine Repurposing on Metabolic Syndrome and Diabetic Complications

**DOI:** 10.3390/nu15112515

**Published:** 2023-05-29

**Authors:** Lu Cai

**Affiliations:** Pediatric Research Institute, Departments of Pediatrics, Radiation Oncology, Pharmacology and Toxicology, The University of Louisville School of Medicine, and Wendy Novak Diabetes Institute, Norton HealthCare, Louisville, KY 40202, USA; lu.cai@louisville.edu

Both obesity and diabetes are global health threats due to their high risk of developing different complications. Therefore, we called this Special Issue, with the purpose of attracting research on both diseases’ underlying mechanisms and the potential interventional applications of certain nutrients and natural products that sufficiently stimulate the body’s defense system such as anti-oxidative stress and anti-inflammation, as well as improvements in insulin signaling (resistance). In order to accelerate translation into clinical use, we also called upon work with FDA-approved medicines or those commonly used in the clinic. At this end, we collected six publications, including five original studies and one review. Although there were only six publications, these covered a human population and risk study, pre-clinical studies with animal models and cell cultures, and a literature review. Therefore, as a famous saying goes, “A sparrow may be small, but it has all its vital organs”.

It is known that metabolic and diabetic diseases are multifactorial, multifaceted, and even intertwined, particularly regarding the interaction of genetic risk and environmental exposure, all of which make understanding the etiopathogenesis of such diseases more complex than originally thought. For instance, the metabolism of betaine, folate, and methionine is interwoven with that of homocysteine in a one-carbon cycle, with a metabolic change in any of these components potentially affecting individual risks of developing metabolic syndrome and diabetes. In this Special Issue, therefore, Lu et al. demonstrated the associations between serum betaine or methyl-metabolizing genetic polymorphisms, and the risk of type 2 diabetes (T2D) in Chinese adults [1]. In their study, they recruited 1565 subjects without T2D at baseline. They followed up with these participants for about 9 years and found that 213 participants developed T2D. An analysis of their serum betaine levels and methylenetetrahydrofolate reductase (MTHFR) gene mutation status revealed that the participants with the highest levels of serum betaine had a lower risk of T2D compared with those with the lowest levels. For *MTHFR* G1793A, participants carrying the heterozygous or homozygous variants (GA and AA, respectively) had the lowest risk of T2D compared with those carrying the normal genotype (GG), while for *MTHFR* A1298C, participants who carried the heterozygous or homozygous variants (AC or CC, respectively) showed significantly decreased risk compared with those without a mutation (AA). The interactions of serum betaine, and the *MTHFR* G1793A or *MTHFR* A1298C genotype were found to synergistically reduce T2D risk. These findings are very informative and important, since except for several previous studies that showed the association of methyl-metabolizing genetic polymorphisms with cancers [2,3], relatively less attention has been paid to the risk of T2D due to these two variables, particularly both together. Therefore, this aspect needs to be paid more attention in future studies.

11β-hydroxysteroid dehydrogenase type 1 (11β-HSD1) acts as a key enzyme in glucocorticoid metabolism, and glucocorticoids can impair glucose-dependent insulin sensitivity. Curcumin is well known as a natural extract with anti-inflammatory and anti-oxidative stress activities. One of the curcumin analogues, H8, was synthesized and able to inhibit 11β-HSD1 and to alleviate insulin resistance in db/db T2D mice [4]. In the study by Chen et al. [5], a preclinical T2D rat model induced by a high-fat diet (HFD) for 8 weeks, followed by an intraperitoneal injection of streptozotocin (HFD/STZ), showed an H8-mediated alleviative effect on non-alcoholic fatty liver disease (NAFLD) in T2D rats. With in vitro and in vivo approaches, they demonstrated that H8 alleviates hepatic steatosis by mechanistically inhibiting 11β-HSD1, which in turn activated the AMP-activated protein kinase (AMPK)/SIRT1 anti-oxidative and anti-inflammatory pathway.

In line with the study by Chen et al. above, systemic inflammation in other organs of individuals with either metabolic syndrome or diabetes are also causative of these organ complications. 12/15-lipoxygenase (LO)-mediated inflammation is also involved in insulin resistance and obesity-associated complications such as kidney dysfunction [6]. The treatment of glomerular mesangial cells cultured in vitro with a metabolite of 12/15-LO, 12(S)-HETE, significantly increased TNF-α, MCP-1, and IL-6 mRNA expression. Urinary albumin excretion was greater in HFD-fed mice than in standard-fat-diet-fed mice, but both their urinary protein and microalbumin amounts were lower in HFD-fed 12/15-LO knockout mice than in WT mice. The levels of TNF-α, IL-6, and MCP-1 in serum and the renal cortex were higher in WT mice than in 12/15-LO knockout mice. ChIP assays showed that 12(S)-HETE increased H3K4me modification in the TNF-α, IL-6, and MCP-1 gene promoters and decreased H3K9me3 modification in the MCP-1 and IL-6 gene promoters. Therefore, this study showed that 12/15-LO may regulate the expression of inflammatory factors in obesity-related glomerular disease via the methylation of histones in the promoter regions of genes encoding inflammatory factors [6]. Additionally, diabetic cardiomyopathy (DCM) is a common diabetic complication. Sun et al. reported that krill oil (KO), extracted from *Euphausia superba* (Antarctic krill), is an alternative source of marine omega-3 fatty acids that could prevent DCM in HFD/STZ-induced T2D mice. The preventive effect of KO on T2D-induced pathogenesis was also associated with its potent inhibitory effect on the NLR family pyrin domain containing 3 (NLRP3) inflammasome by upregulating the expression of Sirtuin 3 (SIRT3) and peroxisome proliferator-activated receptor-γ coactivator 1α (PGC-1α), which are negative regulators of NLRP3 [7].

Except for the common complications mentioned above, a new neuronal complication of diabetes has recently emerged and was recognized as having an Alzheimer’s disease (AD)-like pathology; therefore, some call AD type 3 diabetes (T3D) [8]. Since there are similarities between these two pathogeneses, the pathogenic effects of advanced glycation end products (AGEs), which have been considered the main pathogenic factor of diabetes, on the neuronal system have been explored [9]. Before human neuroblastoma SH-SY5Y cells were treated with AGEs, the addition of diosmetin significantly increased cell viability, prevented AGE-induced oxidative stress, increased amyloid precursor protein and amyloid-β production, and downregulated antioxidant enzyme activities. Diosmin is a flavonoid from citrus fruits, and studies have indicated that diosmin possesses diverse pharmacological activities, including anti-inflammatory, anti-hyperlipidemic, anti-hyperglycemic, and anti-oxidative stress activities. Following oral administration, diosmin is quickly hydrolyzed by enzymes from the intestinal microflora into diosmetin; therefore, diosmetin was directly used in the in vitro study, concluding that the diosmetin protection of cells against AGE-induced neuronal cell injury may have potential in the prevention of diabetic AD or T3D [9].

In compliance with our call for studies on natural products with potential application in individuals with metabolic syndrome and/or diabetes, a review on vitamin B12 deficiency due to chronic use of metformin, which is an FDA-approved and commonly used medicine for T2D in Arab countries, was conducted by Alhaji [10]. Overall, lower serum vitamin B12 levels in patients with T2D are associated with a longer duration and higher dose of metformin use in Arab countries. Therefore, further studies must be conducted to identify patients who may benefit from vitamin B12 supplementation. Finally, the author suggests that routine checking of serum vitamin B12 levels is required in patients with T2D and that clinicians should be provided with the American Diabetes Association recommendations regarding the management of diabetes and its complications [10].
